# Vein in Vain: A Case of Deep Vein Thrombosis Following the Use of the Mynx Closure Device

**DOI:** 10.1155/cric/8650666

**Published:** 2025-11-27

**Authors:** Alexander Yaylayan, Vineet Madishetty, Kevin Schneider, Mohamed Tashani, Jesse Klein

**Affiliations:** HCA Healthcare/USF Morsani College of Medicine GME: Internal Medicine/Interventional Cardiology, HCA Florida Largo Hospital, Largo, Florida, USA

## Abstract

Vascular closure devices (VCDs) have significantly advanced catheter-based interventions by expediting hemostasis following arterial puncture. The Mynx closure device, which uses a sealant consisting of polyethylene glycol (PEG) to facilitate hemostasis, is a widely used tool for arterial closure. Despite their effectiveness, these devices are still associated with complications, including thrombosis and vessel occlusion, which warrant attention. We report a case involving a 58-year-old male with a complex medical history, including atrial flutter and nonischemic cardiomyopathy, who presented with right lower extremity pain following catheter ablation for atrial flutter. Hemostasis was effectively achieved by using the Mynx closure device. Despite successful recovery, the patient developed a DVT within the right lower extremity, involving the femoral and iliac veins which necessitated mechanical thrombectomy. While VCDs such as the Mynx closure device have shown superior benefits in reducing hemostasis time and improving patient comfort, instances of complications such as deep vein thrombosis and vessel occlusion have been reported. The potential for such complications suggests a need for proper training and careful monitoring to minimize risks.

## 1. Introduction

The modified Seldinger technique for arterial puncture and sheath insertion has become the new standard for performing invasive cardiovascular procedures. With the advancement of techniques and devices, more patients with atherosclerotic disease are undergoing these procedures. As the number of cardiovascular procedures continues to rise, the need for effective techniques for arterial closure is becoming more crucial.

Manual compression has long been the gold standard method for closing the arteriotomy site, necessitating careful monitoring and immobilization to be effective [1]. In 1995, the first arteriotomy closure devices (ACDs) were introduced to minimize vascular complications and reduce the time required for hemostasis and ambulation [1]. Vascular closure devices (VCDs) have now become prominent in the field of catheter-based interventions due to their capacity to expedite the healing process immediately following arterial puncture [1]. A range of such devices is currently available on the market, each employing its own distinctive way of achieving hemostasis. Suture-based devices utilize fine needles to deliver sutures through the arterial wall, which are subsequently tied to securely close the puncture, effectively creating a surgical knot within the vessel [2]. Collagen plug devices leverage naturally occurring substances found inside the body to promote clot formation [2]. Lastly, clip-based devices employ small clips or clamps that mechanically seal the puncture by exerting direct pressure [2].

The Mynx closure device works by way of utilizing a balloon catheter to deliver a water-soluble sealant made up of polyethylene glycol (PEG) which works by absorbing blood and fluids around the puncture site via a porous structure followed by swelling to cover the arteriotomy site within the tissue tract [3]. This provides a framework for natural hemostasis to occur with subsequent swelling of the porous structure to up to four times its original size expanding both vertically and horizontally within the tract [3]. Due to the composition of the sealant containing 5% PEG and 95% blood and subcutaneous tissue, the body is effectively able to resorb the sealant within 30 days of deployment from the closure site.

While this device plays a pivotal role in achieving effective hemostasis, particularly in comparison to the gold standard of manual compression, it is imperative to consider the potential complications associated with its use. Despite its benefits, VCDs are not without risk, as they can give rise to complications such as access-site bleeding, thrombosis, arteriovenous fistula formation, dissection, vessel occlusion, pseudoaneurysm formation, and other vascular-related issues [4]. We report a case of a 58-year-old male who developed deep vein thrombosis (DVT) following a catheter ablation procedure, in which hemostasis was achieved using the Mynx VCD.

## 2. Case Presentation

A 58-year-old male with a past medical history of atrial flutter, nonischemic cardiomyopathy, patent foramen ovale closure, a left temporoparietal ischemic stroke requiring mechanical thrombectomy with right-sided residual deficits, history of an ablation, and hyperlipidemia presented with sudden onset of right lower extremity pain. He was hospitalized the month prior to presentation, for a scheduled electrophysiology study with catheter ablation of his atrial flutter.

The procedure was uncomplicated with successful termination of the atrial flutter. The sheaths were removed with subsequent hemostasis obtained via the Mynx hemostasis devices. He was monitored overnight without any postoperative complications and discharged the following day. He had followed up in the outpatient setting approximately 2 weeks after discharge complaining of right lower extremity pain. He reported compliance with his apixaban and attempted to perform conservative measures to help alleviate the pain such as elevating his right lower extremity without improvement in his pain. He was subsequently found to have a right lower extremity DVT with extensive involvement of the femoral and iliac veins. He was referred to have a mechanical thrombectomy of the right lower extremity.

## 3. Past Medical History

The patient has a past medical history of atrial flutter, nonischemic cardiomyopathy, patent foramen ovale closure, a left temporoparietal ischemic stroke requiring mechanical thrombectomy with right-sided residual deficits, a history of an ablation, and hyperlipidemia.

## 4. Management

**Figure 1:**
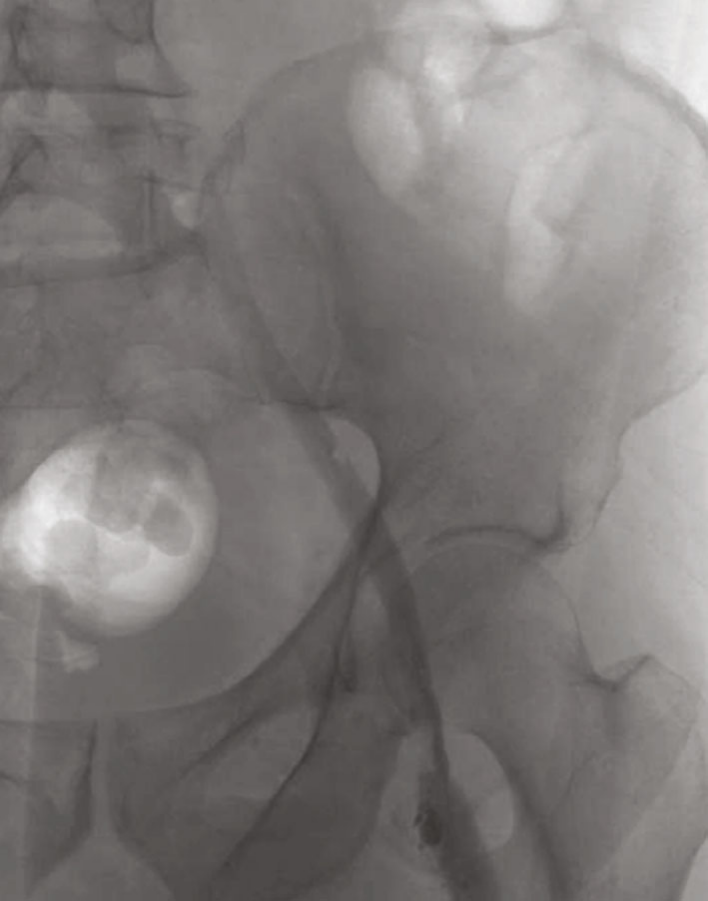
Initial angiogram showing venous stenosis.

**Figure 2:**
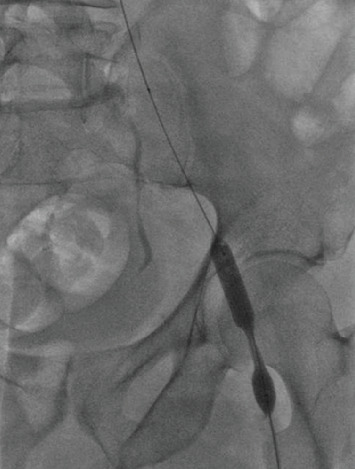
Initial inflation of angioplasty balloon demonstrating stenosis.

**Figure 3:**
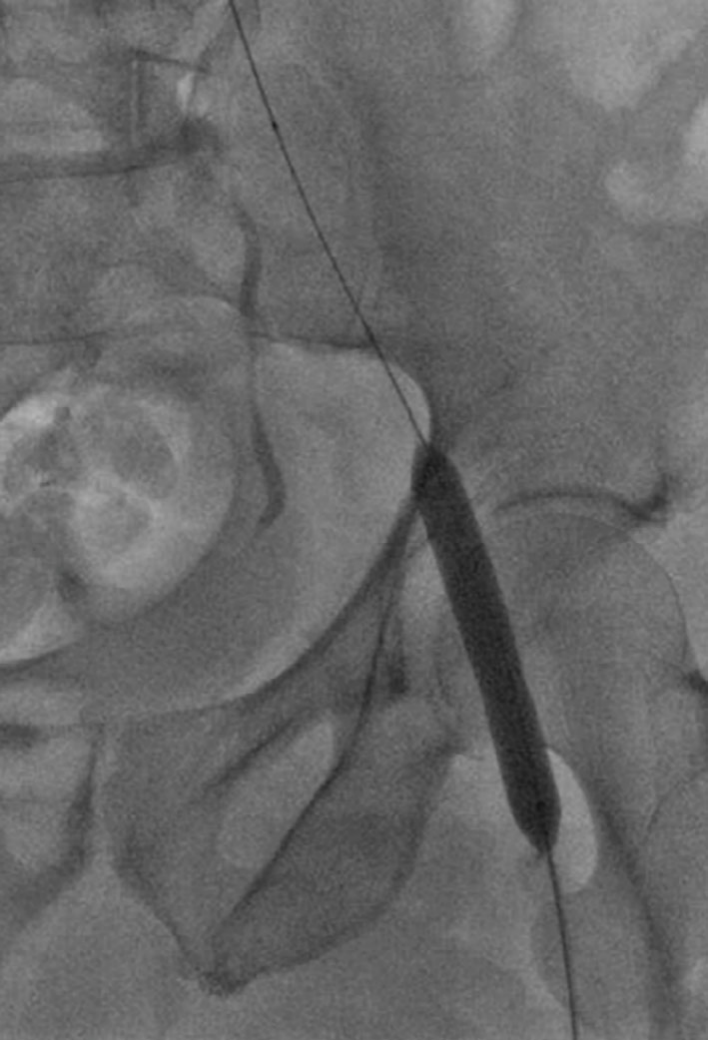
Full angioplasty balloon expansion.

**Figure 4:**
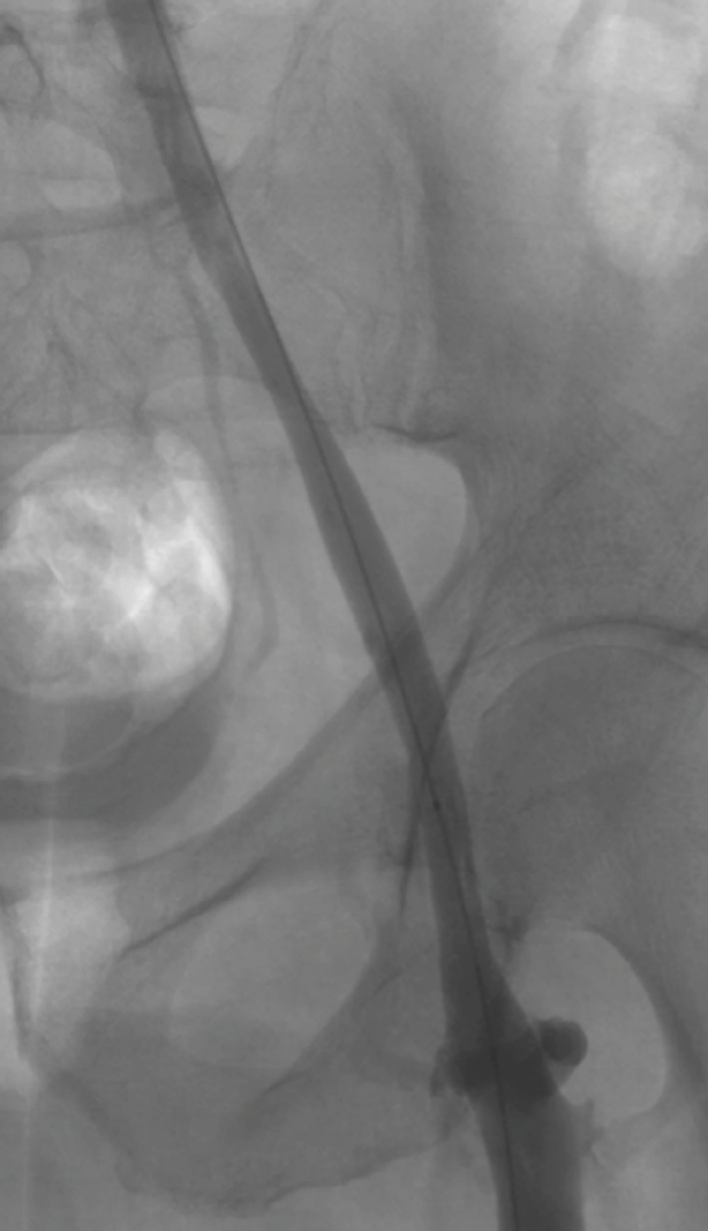
Final angiogram showing resolution of venous stenosis.

Right lower extremity ultrasound revealed a DVT in the femoral vein. He subsequently underwent mechanical thrombectomy of the right lower extremity using the ClotTriever (Inari) device involving the right external iliac vein and right common femoral vein. He was found to have mild-to-moderate external compression of the right iliac external vein approximately 40%–45% with an associated artery; however, there was severe stenosis greater than 90% within the common femoral vein stenosis with mixed etiology of both thrombus and fibrotic webbing. For this reason, the patient's procedure was converted to percutaneous endovascular intervention. The patient had multiple rounds of intravascular ultrasound (IVUS) in the inferior vena cava, right iliac vein, and right femoral vein. There was still significant webbing; therefore, an additional run of venous thrombectomy was completed with the ClotTriever (Inari) device followed by IVUS which resulted in a residual stenosis of 10%–15% with normal blood flow (Figures 1, 2, 3, and 4).

## 5. Discussion

VCDs are gaining popularity due to their ability to reduce the time of hemostasis in comparison to the gold standard of manual compression. The majority of VCDs used today utilize collagen-based or suture–metallic-based technologies to compress the arteriotomy. VCDs have been proven to hasten hemostasis, improve patient comfort, and expedite the time to discharge compared to manual compression [6]. Despite the advantages, there are known complications associated with the usage of VCDs such as access-site bleeding/hematoma formation, thrombosis, arteriovenous fistula formation, dissection, vessel occlusion, septic emboli, and pseudoaneurysm formation [7]. The overall rate of vascular complications with VCDs was measured at 1.5%–9% with serious infection complication rates measuring as high as 5.1% [7–9].

According to the database from the Manufacture and User Facility Device Experience (MAUDE) regarding 236 reports of the Mynx VCDs from July 2015 to June 2021, higher rates of vessel occlusion resulting in limb ischemia (15.8%, *n* = 3/11), hematoma (36.8%, *n* = 7/18), and device embedment (10.5%, *n* = 2/5) were found (*p* ≤ 0.001) [10]. The primary failure mechanisms included device detachment (46%, *n* = 109), unsuccessful deployment (29%, *n* = 68), improper positioning (13%, *n* = 31), and entrapment (3%, *n* = 8) with the majority of incidents (62%, *n* = 146) not having significant clinical impact [10].

Additionally, a systematic search of Embase and MEDLINE was conducted to assess the use of VCDs in antegrade common femoral artery (CFA) and superficial femoral artery (SFA) interventions from inception to October 2019. A total of 24 unique studies were identified in this systematic search, encompassing 4124 vascular closure events across six different VCDs. Clinically significant variations were observed in the complications associated with each device, with a trend toward statistical significance. The VCDs included in this search were Angioseal, Exoseal, Femoseal, Glubran 2, Mynx, and Starclose (3698 CFAs and 426 SFAs). Among the 24 studies, one of which examined patient safety by comparing the Mynx VCD to other approved VCDs, with the primary outcome being any vascular complication, and analyzed a large cohort of 73,124 patients. The findings revealed that the Mynx device carried a significantly higher risk of vascular complications compared to other VCDs (absolute risk, 1.2% vs. 0.8%; relative risk, 1.59; 95% confidence interval [CI], 1.42–1.78; *p* < 0.001); there was also a significantly greater risk of access-site bleeding (absolute risk, 0.4% vs. 0.3%; relative risk, 1.34; 95% CI, 1.10–1.62; *p* = 0.001) and transfusion (absolute risk, 1.8% vs. 1.5%; relative risk, 1.23; 95% CI, 1.13–1.34; *p* < 0.001) [11].

## 6. Conclusion

VCDs have revolutionized postprocedural hemostasis by expediting closure, reducing time to ambulation, improving patient comfort, and potentially decreasing hospital stays. Compared to manual compression, VCDs offer a more efficient means of achieving hemostasis, particularly in patients undergoing catheter-based interventions. However, these advantages still come with risks such as device failure, malpositioning, hematoma formation, and retroperitoneal bleeding. While complications with VCDs are uncommon, it is imperative that the interventionist operating these catheters receive proper training and demonstrate proficiency in their use to ensure safe and effective vessel closure, as severe complications can have life-threatening consequences.

## Data Availability

As a case report, the patient's individual information is not available for review, in accordance with HIPAA compliance. Prior to the development and submission of the manuscript, written informed consent was obtained from the patient.
